# Senescence Reprogramming by MTHFD2 Deficiency Facilitates Tumor Progression

**DOI:** 10.7150/jca.99168

**Published:** 2024-10-28

**Authors:** Ping Wang, Zhou Fang, Wei Pei, Qi Wu, Tingting Niu, Chengyuan Dong, Minkang Wu, Bei Li, Zhijie Gao, Ping Wang

**Affiliations:** 1 Medical College, Anhui University of Science and Technology, Huainan, AnHui, China.; 2 Tongji University Cancer Center, Shanghai Tenth People's Hospital, School of Medicine, Tongji University, Shanghai, China.; 3 Department of Breast and Thyroid Surgery, Renmin Hospital of Wuhan University, Wuhan, Hubei, China.; 4 Department of Pathology, Renmin Hospital of Wuhan University, Wuhan, Hubei, China.

**Keywords:** senescence, tumor, cell cycle, MTHFD2

## Abstract

**Background:** Age is a critical risk factor for cancer, as its incidence and mortality increase with age. However, there is limited understanding of the molecular changes aging induces in tumors.

**Methods:** We explored demographic differences between young and old cancer patients and identified age sixty and above as pivotal in cancer prognosis. Subsequently, we developed an aging-related prognostic model based on genes to assess senescence's impact on aging-associated cancer. Grounded in the coefficients and expression levels of these identified signature genes, a risk score was computed, enabling the classification of collected samples into aging-related high-risk and low-risk cohorts.

**Results:** Our study revealed increased genomic instability and somatic mutations in tumors from older individuals. We also found alterations in carcinogenic signaling pathways, particularly immune responses, inflammatory pathways, and cell cycle arrest in susceptible populations. Single-cell RNA sequencing showed heightened frequencies of exhausted T cells, myeloid cells, and B cells in high-risk cohorts.

**Conclusion:** MTHFD2 emerged as a crucial molecular switch regulating senescence in cancer. Its deletion promoted tumor growth by inducing cell senescence and stimulating the senescence-associated secretory phenotype (SASP) in senescent tumor cells. This highlights the need for tailored methodologies in effective cancer management.

## Background

Aging, a complex and inevitable biological process, involves the gradual deterioration of physiological functions and increased vulnerability to aging-related diseases. In human biology, aging encompasses an intricate interplay of genetic and environmental factors^1^, leading to myriad changes across biological, psychological, and social dimensions. The mechanisms underlying aging are diverse, with theories ranging from programmed genetic sequences to cellular damage accumulation^2^, including DNA damage, mitochondrial dysfunction, and the impact of free radicals. Telomere shortening and epigenetic changes further contribute to this complex process^3^. Aging is the most significant risk factor for cancer, associated with an exponential increase in both incidence and mortality rates among various cancer types^4^. Nevertheless, the intricate correlation between aging and the molecular determinant of cancer remains to be fully characterized.

The intricate relationship between the aging process and cancer signifies a pivotal domain within gerontology and oncology^1^,^3^,^4^. The underlying mechanisms underlying aging and cancer involve a complex interplay of genetic, cellular, and environmental factors. Mechanistically, aging is associated with a range of cellular and molecular changes that contribute to increased cancer susceptibility^4^. These changes, including genomic instability, telomere shortening, epigenetic alterations, and a dysfunctional microenvironment, may contribute to carcinogenesis^3^-^5^. Consequently, we hypothesized that, owing to the variances in selective pressures stemming from changes in tissue environments with age, tumors originating from individuals at different life stages may exhibit distinct molecular landscapes. Consequently, certain molecular alterations may be more or less prevalent in older or younger patients^4^.

Aging influences cellular metabolism, and modifications in metabolic pathways play a significant role in the genesis of cancer^4^,^6^. For instance, a decrease in one-carbon metabolism becomes apparent in the aging process, and the perturbation of this metabolic pathway precipitates tumor proliferation and immune subversion. One-carbon metabolism, an integral biochemical network within cellular physiology, oversees the conveyance and utilization of one-carbon entities for a myriad of cellular processes^7^. This elaborate cascade encompasses a succession of interconnected biochemical reactions indispensable for the biogenesis of nucleotides, amino acids, and other molecules that are imperative for cellular functionality^8^,^9^. Methylenetetrahydrofolate dehydrogenase 2 (MTHFD2), a pivotal enzyme in cellular biochemistry, plays a pivotal role in one-carbon reactions^10^. Positioned at the nexus of one-carbon metabolism, this enzyme catalyzes the conversion of 5,10-methylenetetrahydrofolate to 5,10-methenyltetrahydrofolate, a pivotal juncture in the synthesis of purines and thymidylate, indispensable components of DNA^11^. MTHFD2 has been the subject of exhaustive scrutiny within the domain of cancer research because of its linkage to the metabolic adaptation of tumors. Elevated expression of MTHFD2 is recurrently noted in diverse cancer manifestations due to its overexpression concomitant with heightened cellular proliferation, invasive tendencies, and resistance to chemotherapy^12^. Although the age-related implications of MTHFD2 alterations in the genesis and prognosis of cancer remain to be fully clarified, MTHFD2 has emerged as a promising target for therapeutic interventions^13^.

In this study, we conducted a systematic examination of aging-related disparities in genomic instability, somatic copy number alterations (SCNAs), somatic mutations, pathway modifications, and gene expression across various cancer types^5^,^14^,^15^. We investigated age-correlated indicators in cancers to precisely prognosticate outcomes^16^,^17^. Moreover, MTHFD2 plays a pivotal role as a prognostic factor in cancer, and MTHFD2 loss accelerates aging-like alterations to promote tumor growth^18^-^20^. Ultimately, our research elucidates the significant impact of MTHFD2 as a prognostic determinant in cancer, where MTHFD2 deficiency expedites senescence-like alterations to foster tumor growth.

## Results

### Aging-linked gene signatures forecast cancer prognosis

**Figure 1:**
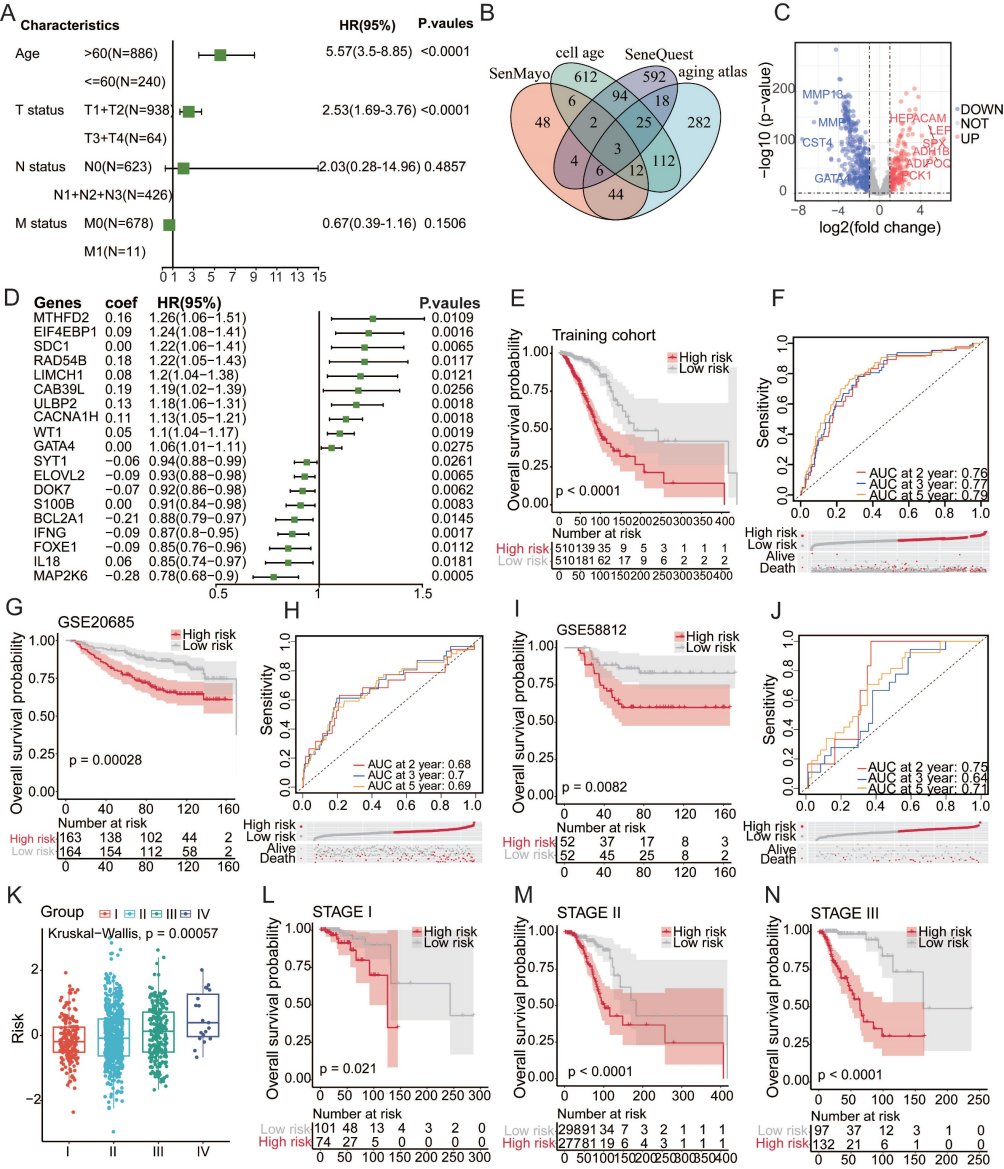
** Aging-linked Gene Signatures Forecast Cancer Prognosis.** (**A**)Univariate Cox regression analysis based on cross-validation and minimum partial likelihood deviation of clinical information. (**B**) Venn diagram of four aging-related gene databases. (**C**) Volcano plot construction using fold-change values and p-adjustment. Red dots represent upregulated genes; blue dots represent downregulated genes; gray dots represent non-significant genes. (**D**) Cox-LASSO regression algorithm identifies genes with the most robust prognostic significance in the aging-related gene set. (**E-F**) Kaplan-Meier analysis of overall survival (OS) curves for high/low-risk subgroups of patients in the training cohort. (**G-J**) Kaplan-Meier analysis of overall survival (OS) curves for high/low-risk groups of patients in the GEO training cohort. (**K-N**) Survival analysis of high/low-risk groups at different stages.

A meticulous analysis was conducted using the clinical data of 1255 patients to study the impact of patient age on various clinicopathological characteristics and prognoses. The findings suggest that surpassing the age of 60 is a consequential determinant of prognosis in cancer patients (**Table 1**). The investigation highlights a significant difference in outcomes among different age groups, specifically emphasizing the heightened vulnerability of individuals aged 60 and above. Within the cohort of 989 patients under the age of 60, an overwhelming majority of 879 individuals showed no cancer recurrence following systematic treatment, highlighting a positive prognosis in this age group. Conversely, among the 266 patients aged over 60, there was a heightened propensity for cancer recurrence, with 57 individuals experiencing relapse despite rigorous systematic treatment. This significant disparity in recurrence rates highlights an age of over 60 years as a key factor affecting cancer prognosis, emphasizing the need for customized interventions specific to age to enhance treatment effectiveness and reduce the increased risks linked to older age^21^. Following the initial analysis, a subsequent univariate Cox regression analysis was performed on the clinical data (*P*<0.05) (**Figure 1A**), revealing compelling insights into the relationships between age and cancer development and prognosis. The findings unequivocally identified age over 60 years as a significant and independent risk factor^20^.

To identify aging-associated gene alterations, we conducted a thorough examination of public databases and relevant literature, compiling a collection of four aging-related gene databases: SenMayo^22^, CellAge, SeneQuest^23^, and the Aging Atlas^1^ (**Figure 1B**). This meticulously assembled collection comprises a total of 1861 genes associated with aging, of which 1794 genes were confirmed to be expressed within our designated training set. We identified 601 aging-related genes by differentially expressed gene (DEG) analysis that distinguished between normal and malignant tissues. The resulting volcano plot vividly depicted this dichotomy, illustrating 200 upregulated genes and 401 downregulated genes within the tumor samples compared to their normal tissue counterparts (**Figure 1C**). Univariate Cox regression and least absolute shrinkage and selection operator (LASSO) regression algorithms were used to identify genes with the most robust prognostic significance within the aging-related gene set (**Figure 1D**). Grounded in the coefficients and expression levels of these identified signature genes, a risk score was computed, enabling the classification of collected samples into aging-related high-risk and low-risk cohorts. Prognostic analysis revealed a stark divergence, with the high-risk group showing a significantly poorer prognosis (*P* < 0.0001), as illustrated in **Figure 1E**. The high-risk group had a significantly higher rate of death than the low-risk group. The model's predictive performance was evaluated using the area under the curve (AUC) for 1-, 3-, and 5-year overall survival (OS), which were 0.77, 0.67, and 0.65, respectively. To validate the robustness of the findings, GSE20685 and GSE58812 served as independent verification cohorts. Coherently, samples in the validation cohorts, stratified into high- and low-risk groups based on the risk scoring method derived from the training set, displayed a more adverse prognosis in the high-risk category, accompanied by a higher incidence of mortality (**Figure 1F-G**). Further exploration of prognostic status among patients at different stages ensued. The findings indicated that the senescence-related signature score could accurately predict the prognosis of patients at stages I, II, and III. Patients in the high-risk category exhibited a more unfavorable prognosis (**Figure 1H-K**). These findings provide valuable insights into the nuanced relationship between aging and cancer outcomes, prompting further exploration of senescence-tailored therapeutic approaches for more effective cancer management in older populations.

### Aging-associated mutational patterns in cancer

**Figure 2:**
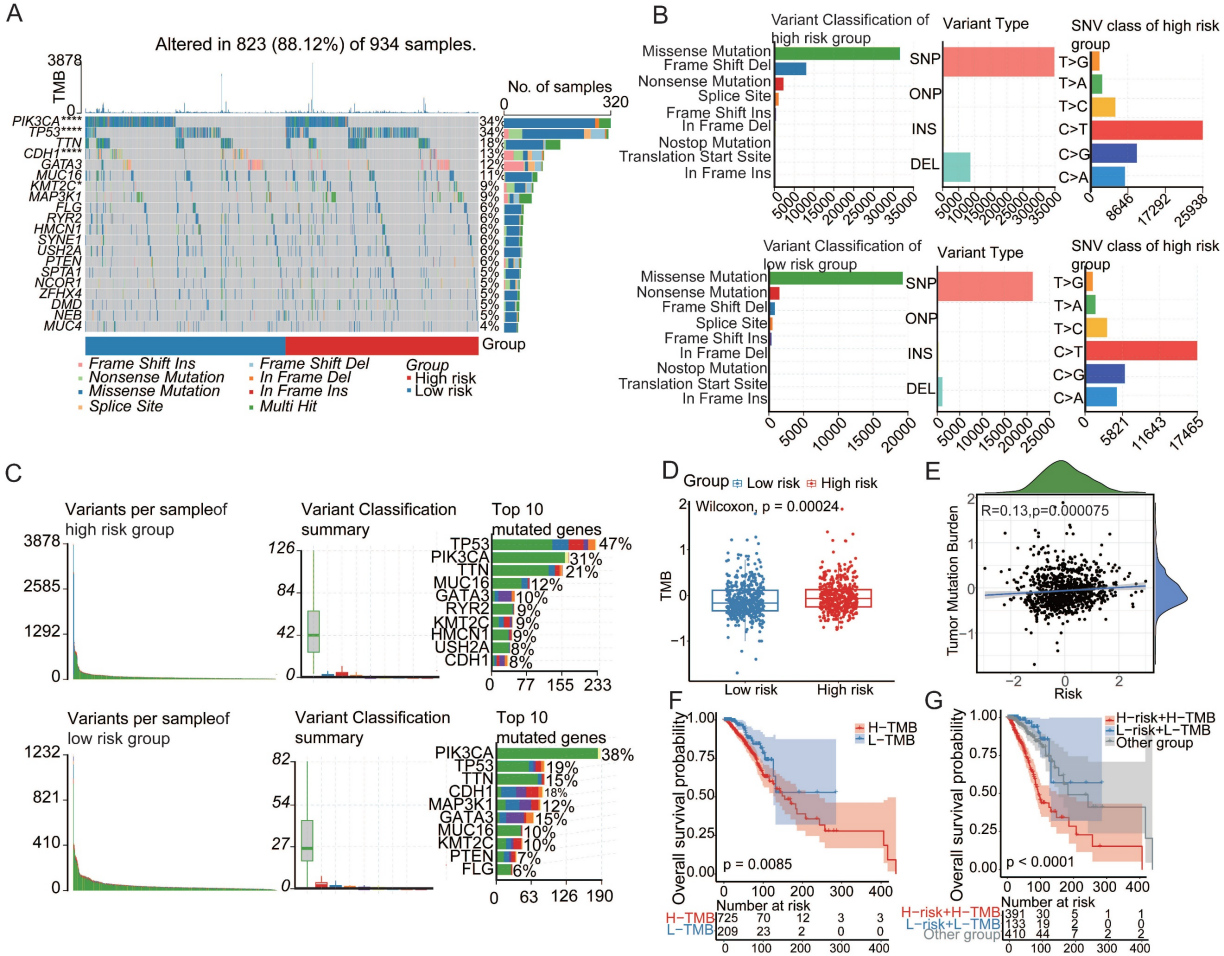
** Aging-associated mutational patterns in cancer.** (**A**) Comparison of mutation profiles between high and low-risk groups. (**B-C**) Summary plots of the cohort displaying the distribution of mutated strains based on mutation type and SNV classification, with stacked bar graphs showing the top 10 mutated genes. (**D**) Boxplot illustrating the correlation between risk scores and TMB in the breast cancer cohort. (**E**) Correlation between risk scores and TMB levels. (**F**) Kaplan-Meier survival analysis of TMB, risk scores, and OS in the breast cancer cohort. (**G**) Kaplan-Meier survival analysis of TMB and OS in the TCGA breast cancer cohort.

Numerous investigations have consistently highlighted the pivotal role of mutational patterns in tumorigenesis. In this study, a comparative analysis was performed to examine the mutational landscape across distinct patient risk stratifications. Remarkably, significant disparities were observed in the mutation status of *PIK3CA*, *TP53*, and *CDH1* between the low- and high-risk cohorts^15^. The prevalence of *TP53* mutations notably increased within the high-risk demographic group, hinting at its potential involvement in the progression of cancer influenced by the aging process^24^ (**Figure 2A**). A meticulous examination of variant classification revealed differences between these two cohorts (**Figure 2B**). *PIK3CA*, *TP53*, and *CDH1*, which are recognized as somatic driver mutations and small insertions/deletions (indels), have previously been implicated in the course of cancer evolution^25^,^26^. The observed variances in frameshift deletion mutations, ranking second in the high-risk category and surpassing nonsense mutations, and conversely in the low-risk category, may be attributed to aging-associated transformations. These findings emphasize the need for thorough investigations to uncover the effects of aging on these mutational patterns and their potential contributions to the development of cancer. Such revelations hold promise for a more nuanced understanding of the intricate interplay between aging and the molecular foundations of cancer development.

Tumor mutation burden (TMB), a robust quantitative metric for assessing mutation levels^27^, revealed a significantly elevated TMB within the high-risk group (**Figure 2D**). Notably, a positive correlation emerged between the risk score and TMB (**Figure 2E**), accentuating their mutual interdependence^29^. Prognostic analysis further elucidated the clinical relevance of the TMB, revealing that increased TMB correlated with an unfavorable prognosis^30^,^31^ (**Figure 2F**). To determine the potential synergistic impact of the TMB on prognosis, a pioneering stratified prognostic analysis amalgamating the two parameters was conducted^28^,^32^. Intriguingly, this analysis revealed an augmented prognostic predictive capacity within the training cohort. Specifically, patients with elevated TMB demonstrated a strong association with an inferior prognosis, while those with low TMB exhibited a more favorable prognosis (*P*=0.0085) (**Figure 2G**). These findings underscore the significance of incorporating TMB assessment for a comprehensive understanding of prognostic dynamics in cancer. This approach offers invaluable insights for personalized therapeutic strategies based on mutational profiles.

### Aging-associated molecular alterations in oncogenic signaling pathways

**Figure 3:**
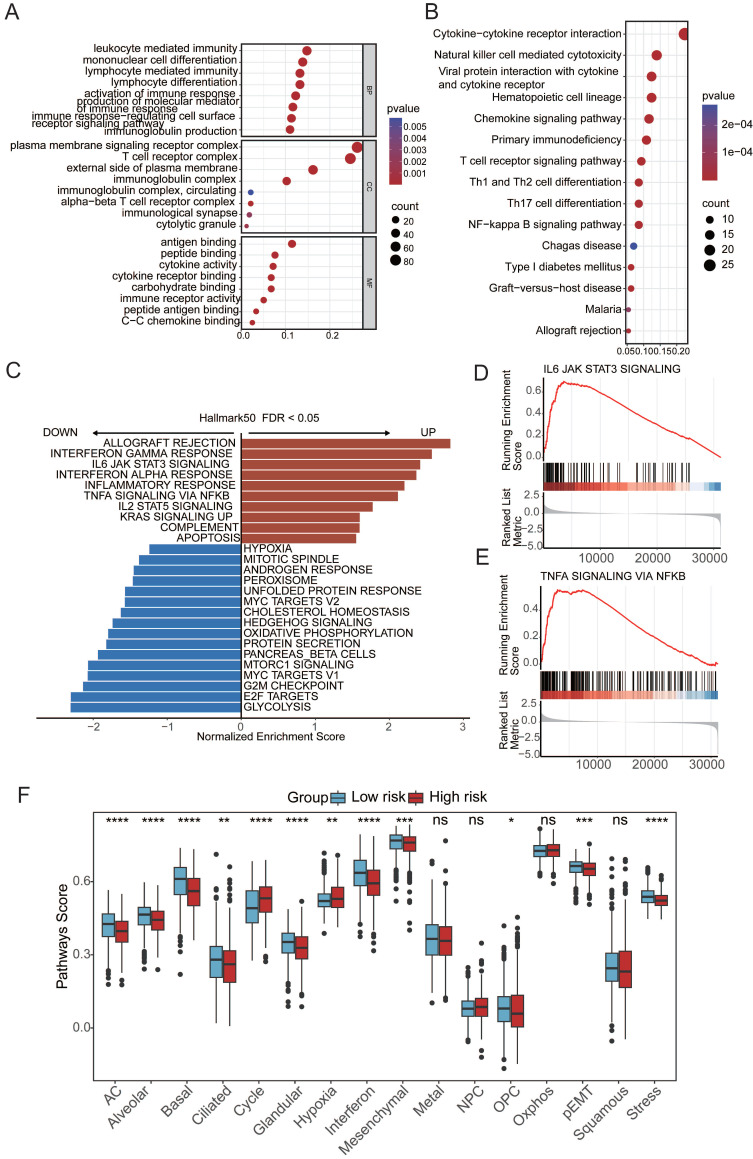
** Aging-associated molecular alterations in oncogenic signaling pathways.** (**A**) GO enrichment analysis of upregulated aging-related DEGs. (**B**) KEGG pathways of upregulated aging-related DEGs. (**C-E**) Gene set enrichment analysis (GSEA) of aging-related prognostic models. (**F**)Boxplot of label scores for 16 cancer cell states in high/low-risk groups based on GSVA scores. Paired two-sided Wilcoxon test.

To elucidate the mechanistic link between aging and cancer, we explored the underlying pathways through Gene Ontology (GO) and Kyoto Encyclopedia of Genes and Genomes (KEGG) analyses based on differentially expressed genes (DEGs) between patients in the high- and low-risk groups. The GO and KEGG enrichment analyses revealed a distinct enrichment profile in the high-risk group, emphasizing immune responses such as leukocyte-mediated immunity, mononuclear cell differentiation, plasma membrane signaling receptor complex, T-cell receptor complex, antigen binding, and immune receptor activity (**Figure 3A-B**). Additionally, gene set enrichment analysis (GSEA) revealed the aggregation of gene sets related to the IFN-γ and IFN-α response and inflammation within the high-risk group, indicating a potential association between these pathways and aging-induced cancer progression (**Figure 3C**). These findings provide valuable insights into the molecular underpinnings of the impact of aging on cancer development, emphasizing the intricate involvement of immune-related processes and signaling pathways. To comprehensively assess molecular functional disparities between patients in the high- and low-risk groups, 16 cancer-related pathway activities were calculated^33^,^34^. This study revealed distinctive patterns of cell cycle dynamics, known for their impact on tissue regeneration, function, inflammation, and tumorigenesis. Specifically, patients in the high-risk group exhibited elevated cycle signature scores, indicative of increased activity within the cell cycle^35^ (**Figure 3F**). This observation suggests a potential association between heightened cell cycle activity and an elevated risk profile, offering valuable insights into the potential drivers of cancer progression influenced by aging^35^.

### Aging-associated alterations in the tumor microenvironment

**Figure 4:**
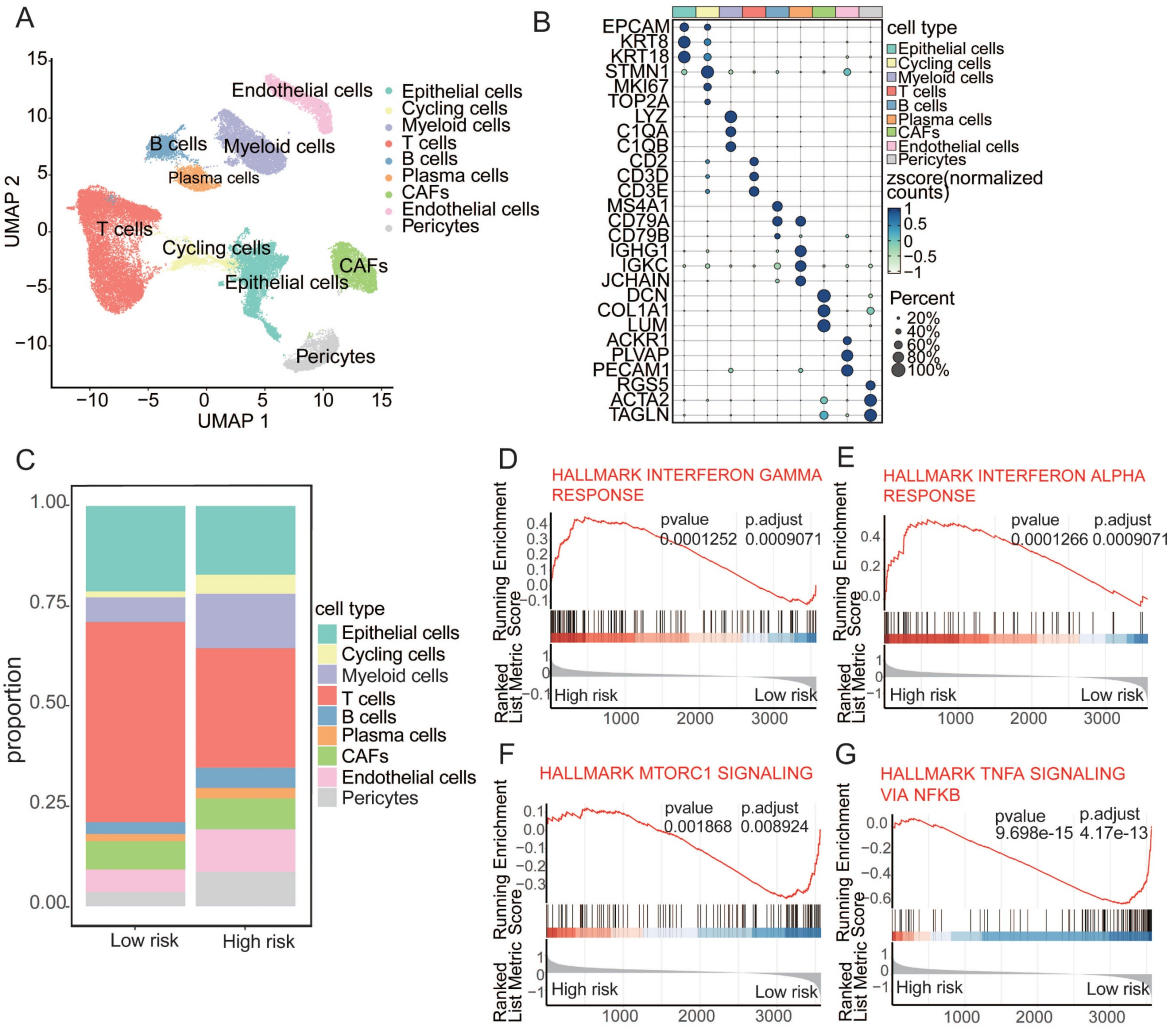
** Aging-associated alterations in tumor microenvironment**. (**A**) UMAP plot depicting major cellular subgroups in cancer. (**B**) Bubble heatmap displaying expression levels of specific feature genes in cancer. The size of the circles represents the proportion of expressing cells, colored based on standardized expression levels. (**C**) Relative proportions of different cell types in high/low-risk tumors. (**D-G**) GSEA analysis of T cells in high/low-risk groups.

In an endeavor to meticulously investigate the distinct cellular compositions of high- and low-risk patients, we collected publicly available single-cell RNA sequencing (scRNA-seq) data paired with bulk RNA-seq data from cancers. By integrating data from 24 samples with paired bulk and scRNA-seq information, we employed the mutual nearest neighbor (MNN) algorithm to mitigate batch effects. Rigorous quality control measures were applied to each individual sample, enabling the depiction of the cancer cellular landscape at single-cell resolution using uniform manifold approximation and projection (UMAP) visualization (**Figure 4A**). Using canonical lineage markers, we accurately annotated each cell population, which included epithelial cells, cycling cells, myeloid cells, T cells, B cells, plasma cells, cancer-associated fibroblasts (CAFs), endothelial cells, and pericytes (**Figure 4B**). For example, the expression of specific markers, such as *CD79A*, *CD79B*, and *MS4A1* for B cells and *EPCAM*, *KRT8*, and *KRT19* for epithelial cells, facilitated precise cell type identification^36^,^37^. The analysis of cellular subpopulations revealed distinctive compositions in the high- and low-risk groups, with significantly lower T-cell levels but higher frequencies of cycling cells, myeloid cells, and B cells in the high-risk group (**Figure 4C**). Further investigation of the functions of T cells within the high- to low-risk comparison revealed upregulation of the IFN-γ and IFN-α response in these T cells. In contrast, the low-risk cohort exhibited a downregulation of mTORC1 signaling and TNFα signaling via NF-κB (**Figure 4D-G**). These findings suggest that aging-driven alterations in the tumor microenvironment contribute to cancer susceptibility^38^.

### MTHFD2 as an aging-associated factor in cancer

**Figure 5:**
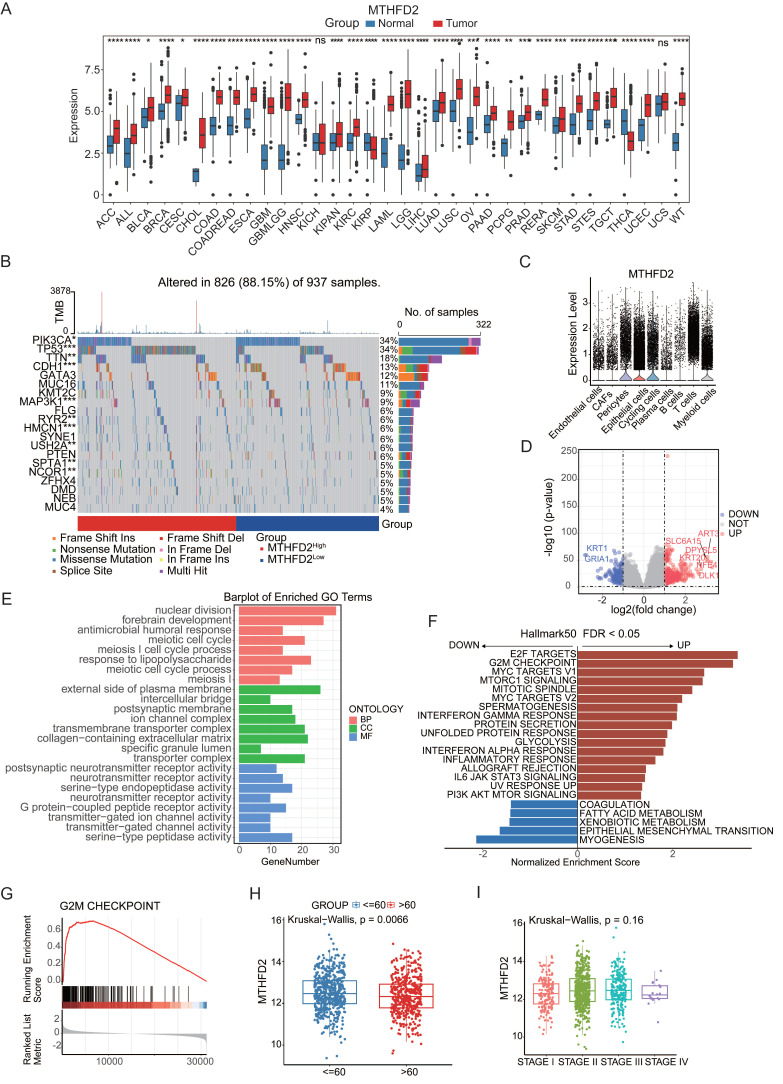
** MTHFD2 as an aging-associated factor in cancer.** (**A**) Differential expression of MTHFD2 in different cancer types based on pan-cancer analysis. (**B**) Oncoplot displaying the somatic landscape of the tumor cohort. Genes are sorted by their mutation frequency, and samples are sorted by MTHFD2 expression, as indicated by the color bar (bottom). The side bar graph shows the -log10-transformed q values estimated using MutSigCV. The waterfall plot shows the mutation information for each gene in each sample. Color annotations for various cancer types are displayed at the bottom. The barplot above the legend shows the number of mutations per sample. (**C**) Boxplot showing the expression levels of MTHFD2 in tumor tissues compared to normal cells. (**D**) Volcano plot constructed using fold-change values and p-adjusted values. Red dots represent upregulated genes; blue dots represent downregulated genes; gray dots represent non-significant genes. (**E-G**) GO and GSEA analysis of differentially expressed genes in MTHFD2 high-expressing and low-expressing cohorts. (**H**) Expression of MTHFD2 in different age groups. (**I**) Expression of MTHFD2 in different staging groups.

In the aforementioned studies, MTHFD2 emerged as both a factor that accentuates aging vulnerability and a protective factor against cancer progression. Consequently, MTHFD2 was chosen for a more in-depth exploration of its intricate role in aging-related cancer. To comprehensively describe the expression of MTHFD2 across a spectrum of tissues and its correlation with clinicopathological features in cancer patients, we conducted an exhaustive exploration employing an online database. MTHFD2 was markedly upregulated in 31 tumors compared with 2 tumors (**Figure 5A**). The discernible expression patterns of MTHFD2 were found to be intricately linked with modifications in ten genes, including *TP53*, *CDH1*, and* MAP3KI*, whereas the mutation status significantly differed between the high- and low-expression groups of MTHFD2 (**Figure 5B**). Single-cell analysis provided additional insights, showing that MTHFD2 was expressed mainly in T cells and myeloid subsets (**Figure 5C**). These simultaneous alterations in gene expression provide invaluable insights into the potential functions of MTHFD2. We conducted GO and GSEA analyses of the differentially expressed genes in the *MTHFD2* high- and low-expression cohorts. These results indicated that MTHFD2 participates in nuclear division and meiotic cell cycle processes, indicating that MTHFD2 regulates cell cycle dynamics (**Figure 5E-G**). GSEA further confirmed this finding, revealing upregulated pathways, such as E2F targets and G2M checkpoints (**Figure 5H**), which are integral to mechanisms regulating the cell cycle. Given the pivotal role of the cell cycle in preserving cellular homeostasis and its intricate linkage to the aging process^39^, our findings suggest that MTHFD2 may contribute to aging-related modifications involved in cell cycle regulation. Subsequently, by analyzing the expression level of MTHFD2 between the young and aging groups, it was found that MTHFD2 expression was higher in the young group (**Figure 5I**), which further confirmed that MTHFD2 may be involved in aging-related modifications.

### MTHFD2 loss drives senescence-like alterations to foster tumor growth

**Figure 6:**
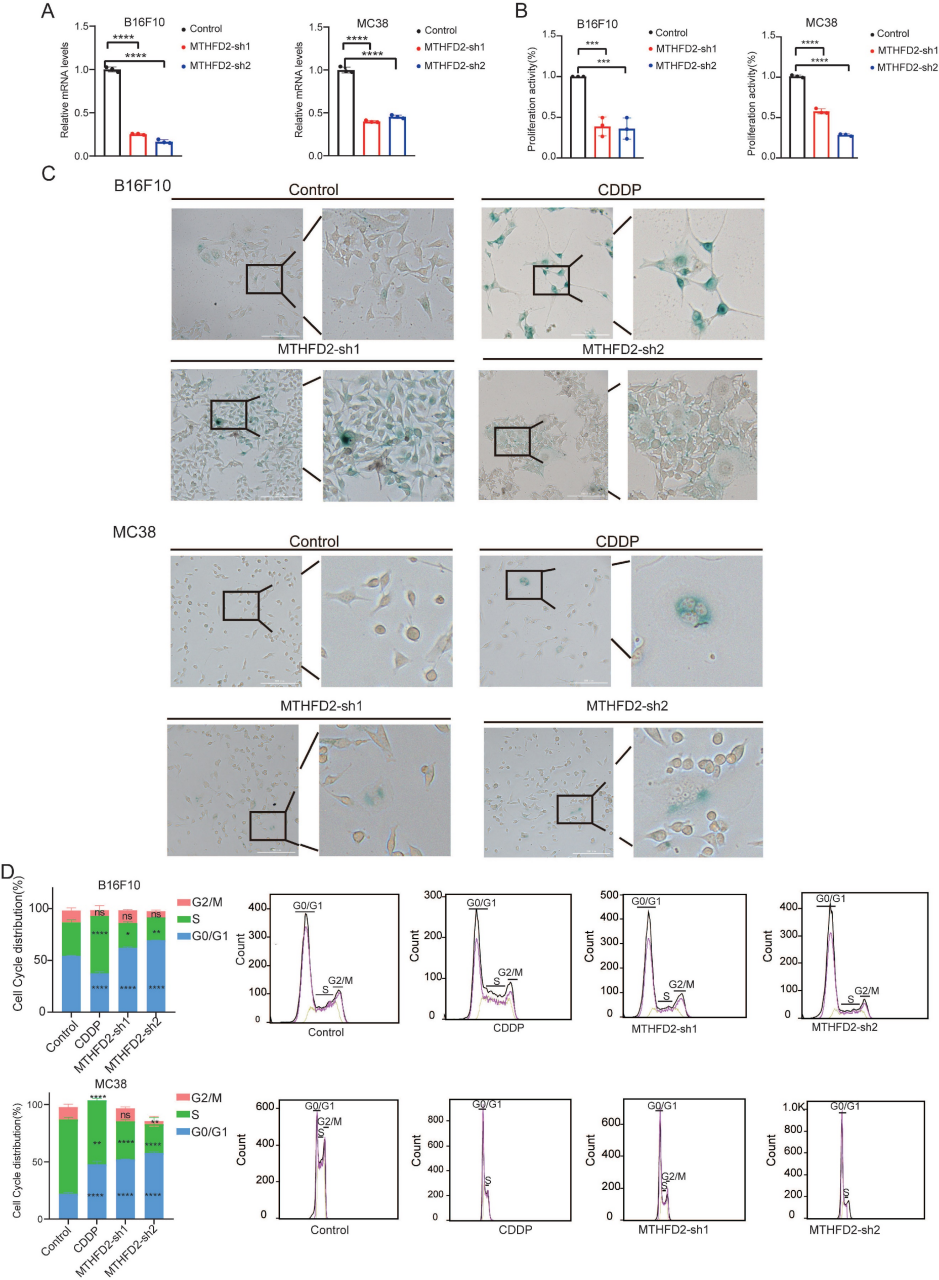
** MTHFD2 loss drives cellular senescence**. (**A**) RT-qPCR analysis of *MTHFD2* knockdown (one-way ANOVA test). (**B**) Growth rate of *MTHFD2* knockdown cells (one-way ANOVA test). (**C**) β-galactosidase staining level in *MTHFD2* knockdown group. (**D**) Flow cytometry analysis of cell cycle in *MTHFD2* knockdown cells.

**Figure 7:**
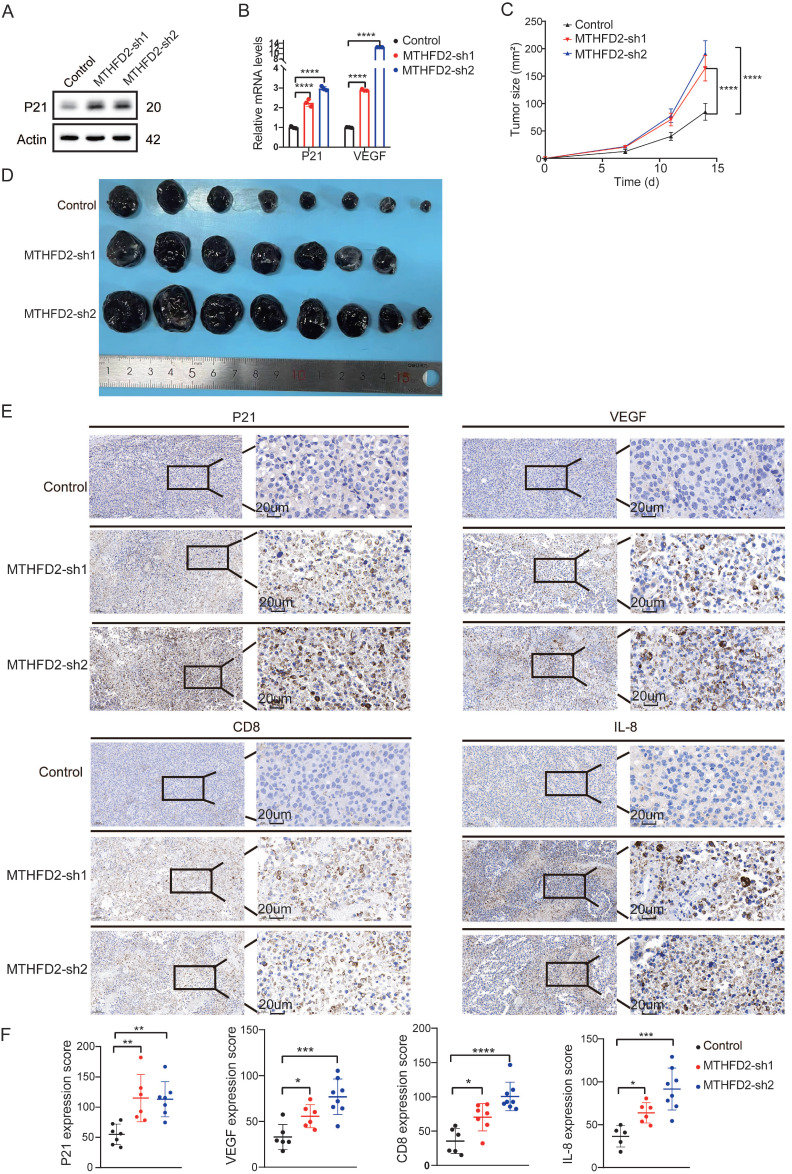
** MTHFD2 loss drives aging-like alterations to foster tumor growth.** (**A**) Protein level of P21 in *MTHFD2* knockdown group. (**B**) mRNA levels of P21 and VEGF in *MTHFD2* knockdown group (one-way ANOVA test). (**C**) Tumor growth curve of C57BL/6J mice injected with *MTHFD2* knockdown B16F10 cells (two-way ANOVA test). (**D**) Photographs of dissected tumors (n ≥ 6). (**E-F**) Immunohistochemical staining in tumor tissues with *MTHFD2* knockdown. A significance level of *P*<0.05 indicates statistical significance. In the Figures, *P<0.05, **P<0.01, ***P<0.001, ****P<0.0001, and "ns" indicates no statistical significance.

In our meticulous exploration of the intricate interplay between MTHFD2 and tumor growth during the aging process, we initiated *MTHFD2* knockdown experiments employing B16F10 and MC38 cells as our experimental model^40^ (**Figure 6A**). Strikingly, we observed notable metamorphoses in cell morphology within the *MTHFD2* knockdown group, which appeared enlarged and flattened in shape, with increased cytoplasmic granularity, vacuolization, and altered nuclear morphology. Simultaneously, the levels of β-galactosidase, which serves as a biomarker for senescence, were measured in cells subjected to *MTHFD2* knockdown and CDDP (Cisplatin) treatment (positive control)^41^,^42^ (**Figure 6C**). Moreover, the rate of cell proliferation significantly decreased under *MTHFD2* knockdown conditions (**Figure 6B**). Additionally, the perturbation of the cell cycle in B16F10 and MC38 cells was meticulously examined through flow cytometry, which revealed a pronounced blockade in the G0/G1 phase within the *MTHFD2* knockdown group (**Figure 6D**). This finding strongly suggested a potential link between MTHFD2 manipulation and impediments in the progression of the cell cycle. Concurrently, the mRNA and protein levels of P21, a pivotal checkpoint in the cell cycle, were increased in the *MTHFD2* knockdown group (**Figure 7A**). Consistent results confirmed the presence of senescence-associated secretory factors such as VEGF in the *MTHFD2* knockdown group (**Figure 7B**). We investigated this possibility by employing B16F10 cells with *MTHFD2* knockdown in a subcutaneous tumor model. In contrast, *MTHFD2* knockdown promoted faster tumor growth (**Figure 7C-D**). Immunohistochemical analysis revealed significantly greater P21, VEGF, and IL-8 staining in tumor tissues from the *MTHFD2* knockdown group than in those from the control group. Additionally, there was a notable increase in CD8^+^ T-cell staining (**Figure 7E-F**). This multifaceted approach not only underscores the potential involvement of MTHFD2 loss in propelling a senescent state but also highlights its potential significance in modulating tumor behavior.

## Discussion

Aging, an intricate biological process, is intricately intertwined with increased susceptibility to senescence-related diseases, notably cancer. Our detailed examination of senescence-related disparities across diverse cancer types revealed age to be a pivotal determinant of cancer prognosis, with individuals aged 60 years and older exhibiting increased vulnerability. By summarizing four different aging-related databases and using the Cox-LASSO algorithm, we obtained aging-associated gene alterations and aging-related risk scores, coupled with the integration of the TMB and exploration of oncogenic signaling pathways, which enriches our understanding of the molecular intricacies shaping cancer outcomes. The dissection of the cellular landscape within different risk groups reveals the complexities of the tumor microenvironment, providing a nuanced perspective. We identified MTHFD2 as both an aging risk factor and a protective factor in aging-related cancer.

By analyzing the cell composition of the high- and low-risk groups, it was found that the T-cell composition of the high-risk group was significantly lower than that of the low-risk group, and T-cell exhaustion plays a pivotal role in rendering older individuals vulnerable to infections and cancer^43^. This decline is responsible for elevated susceptibility to infection and cancer^44^. Further investigations into the functions of T cells within the high- to low-risk group revealed the upregulation of the IFN-γ and IFN-α response in these T cells, and the roles of IFN-γ and IFN-α are complex. While they contribute to immune surveillance against cancer, persistent activation or dysregulation of these interferons may also play a role in chronic inflammation, which is a hallmark of aging and a risk factor for cancer.

MTHFD2 is a crucial enzyme involved in cellular metabolism, particularly in the folate metabolic pathway. This enzyme plays a profound role in nucleotide synthesis, which is essential for DNA replication and cellular proliferation. MTHFD2 catalyzes the conversion of 5,10-methylenetetrahydrofolate to 5,10-methenyltetrahydrofolate, an essential step in the synthesis of purines, amino acids, and other important molecules within the cell. MTHFD2 has garnered attention for its involvement in various cellular processes linked to cancer progression. Multiple investigations have underscored the propensity for increased MTHFD2 expression across diverse cancer types, including breast, lung, colorectal, and pancreatic malignancies^18^. Notably, MTHFD2 is preferentially upregulated in undifferentiated or poorly differentiated tumors^45^, suggesting its cancer-specific expression pattern. In addition to its metabolic functions, MTHFD2 has been implicated in promoting cancer immune evasion. By steering the folate cycle toward the maintenance of adequate UDP-GlcNAc levels, MTHFD2 propels the O-GlcNAcylation of cMYC, thereby bolstering cMYC stability and PD-L1 transcription^46^. Nevertheless, the functional repertoire of MTHFD2 is intricate and multifaceted. Although MTHFD2 is primarily expressed within the mitochondria, it is also present in the nucleus. TH9619 effectively inhibits both the dehydrogenase and cyclohydrolase activities of MTHFD1/2. MTHFD1, found in the cytoplasm, shares similar functions with MTHFD2. TH9619 inhibits MTHFD1 activity, preventing the incorporation of formate produced by the mitochondria into dTMP. It selectively targets nuclear MTHFD2 without affecting mitochondrial MTHFD2, leading to an overflow of formate from the mitochondria. Consequently, cancer cells undergo cell death despite high MTHFD2 expression^47^. In addition, recent studies have indicated that MTHFD2 inhibition induces apoptosis solely in UQCR11-null cells, while it does not affect UQCR11-intact cells^48^. This suggests that the apoptotic effect of MTHFD2 inhibition is specific to cancer cells. Inversely, under conditions of oxygen deprivation or impaired electron transport chain activity, MTHFD2 maintains a substantial supply of NADH through the promotion of serine catabolism, leading to cell death. In hypoxic cells with compromised respiration, inhibition of MTHFD2 partially restores NADH levels and facilitates cell proliferation^45^. These findings suggest a potential anticancer role for MTHFD2. Our findings reveal that deletion of MTHFD2 contributes to cellular senescence. Similarly, prior research has revealed that the colocalization of MTHFD2 with DNA replication sites in the nucleus promotes cell cycle progression. Deletion of MTHFD2 leads to S-phase cell cycle arrest and fosters a senescence-like state^49^. Additionally, MTHFD2 is expressed in developing embryos but is notably absent in most healthy adult tissues, including proliferating ones^50^. This observation implies a decrease in MTHFD2 expression with age. MTHFD2 deletion increases the infiltration of CD8^+^ T cells in B16F10 tumors. The potential mechanisms are manifold. Initially, senescent cancer cells manifest hyperploidy, rendering them genomically unstable and enabling the presentation of tumor antigens to activate immunosurveillance, which entails the recruitment of immune effectors such as B, NK, NKT, and T cells^51^,^52^. Furthermore, the senescence-associated secretory phenotype (SASP) factor VEGF fosters angiogenesis, thereby facilitating T cell infiltration. Nonetheless, the buildup of senescent cells in tumor-bearing mice precipitates T-cell exhaustion^53^. For instance, IL-8, elevated in MTHFD2-depleted cells, augments PD-1 expression in CD8^+^ T cells, leading to T-cell exhaustion. Therefore, the deletion of MTHFD2 facilitates the infiltration of CD8^+^ T cells into tumors but compromises their functional activity. Hence, we propose a novel perspective suggesting that MTHFD2 exerts its anticancer effect by retarding the aging process.

However, some limitations are noted in this study. First, numerous studies have demonstrated that aging is a significant contributor to the development and progression of cancer. Based on data analysis related to breast cancer and animal experiments, this study revealed that downregulation of MTHFD2 can promote cellular senescence, thereby accelerating tumor growth. As such, our study may lack sufficient novelty. Second, the mechanisms involved in this study were not further investigated. For instance, we did not explore how downregulation of MTHFD2 promotes cellular senescence and its impact on tumor immunity during this process. The prognostic role of MTHFD2 in cancer patients requires validation in additional cohorts to confirm its prognostic significance. Finally, the exact effect of MTHFD2 downregulation on the tumor microenvironment remains unclear due to the lack of single-cell sequencing or multi-parameter flow cytometry analyses.

In summary, the process of aging leads to heightened genetic mutations within tumors, triggers the activation of pathways essential for tumor proliferation and spread, and promotes an immunosuppressive microenvironment. The identification of MTHFD2 as a suppressor in tumors associated with aging implies that maintaining its expression and functionality may offer a potential therapeutic approach for addressing such tumors^54^,^55^. Future research directions for MTHFD2 in the diagnosis and treatment of elderly cancer patients could focus on several key areas. These include investigating MTHFD2 as a diagnostic and prognostic biomarker by examining its expression patterns across different cancer types and age groups and analyzing its relationship with patient outcomes. Additionally, understanding the molecular mechanisms of MTHFD2 in cellular metabolism and immune regulation, particularly in the context of aging-related metabolic changes and T-cell functionality, will be crucial. Developing specific MTHFD2 targeting drugs and assessing their efficacy and safety, both as standalone treatments and in combination with existing therapies, is another important avenue^56^. Moreover, focusing on clinical trials involving elderly cancer patients and studying then age-related expression and function of MTHFD2 can provide insights into its role in slowing down aging-associated cancer progression and improving therapeutic outcomes for this population^57^.

## Materials and Methods

### Datasets

The clinical pathological information from 1255 breast cancer patients treated at the Breast and Thyroid Surgery Department of Wuhan University People's Hospital between 2008 and 2016. RNA-sequencing expression matrix and clinical information of breast cancer samples and para-cancerous tissues were downloaded from the Cancer Genome Atlas (TCGA) database on UCSC Xena (https://xena.ucsc.edu/). Two additional independent datasets (GSE20685 and GSE58812) and single-cell RNA-seq data and bulk RNA-seq data 24 of breast tumors (GSE176078) were obtained from the GEO database (https://www.ncbi.nlm.nih.gov/geo/). Aging-related genes were collected from SenMayo (https://genomics.senescence.info/cells/), cell age (https://genomics.senescence.info/cells/), SeneQuest (http://Senequest.net), and the Aging Atlas (https://ngdc.cncb.ac.cn/aging/index). Somatic mutation data were downloaded from the Genomic Data Commons (GDC) (https://portal.gdc.cancer.gov/). The somatic mutation data, sorted in the form of Mutation Annotation Format (MAF), were analyzed and used to calculate the Tumor Mutation Burden (TMB) using the R package maftools.

### Construction and validation of aging-related prognostic signature

To identify genes associated with aging and construct a prognostic signature, we conducted two types of regression analyses: univariate Cox -LASSO regression. Through this analysis, we were able to identify 19 genes: *MTHFD2*, *EIF4EBP1*, *SDC1*, *RAD54B*, *LIMCH1*, *CAB39L*, *ULBP2*, *CACNA1H*, *WT1*, *GATA4*, *SYT1*, *ELOVL2*, *DOK7*, *S100B*, *BCL2A1*, *IFNG*, *FOXE1*, *MAP2K6*. These genes were used to develop an aging-related prognostic model based on these genes. To categorize breast cancer patients, we calculated the risk score for each patient in the training set using the following formula:

Risk score=∑ni=∑(Coefi*xi)

The cancer patients were classified into high-risk and low-risk groups based on the median of their risk scores^58^,^59^. The R package survivalROC was utilized to estimate the predictive sensitivity of the risk score. The efficacy of the model was assessed in the validation set using the same coefficient and cutoff values that were employed in the training set.

### Biological functional analysis between high/low-risk group patients

The DESeq2 R package was utilized to analyze differentially expressed genes (DEGs). DEGs were identified using a cutoff of an adjusted p-value of less than 0.05 and a fold change of |Log2| greater than 1. Gene set enrichment analysis (GSEA) was conducted using the clusterProfiler R package. Fisher's exact test was employed to determine significant indicators, with a false discovery rate (FDR)-corrected p-value threshold of less than 0.05. Single-sample gene set enrichment analysis was performed using the GSVA R package^60^. Gene signatures of recurrent cancer cell states were obtained from a previous study.

### Cell culture

We generated the B16F10/MC38-MTHFD2 cell line through knockdown experiments in B16F10/MC38 cells. We targeted two sites of the MTHFD2 gene and obtained two knockdown cell lines, which we named B16F10/MC38-MTHFD2 sh1 and B16F10/MC38-MTHFD2 sh2, respectively. For our knockdown experiments, we used B16F10/MC38-NC as the negative control. All cells were cultured in Dulbecco's modified Eagle's medium (DMEM) supplemented with 10% fetal bovine serum and 1% penicillin/streptomycin at 37°C in 5% CO_2_. The shRNA sequences are listed below:

MTHFD2-sh1: GCTCATGAAGAACACCATTAT

MTHFD2-sh2: CGGTCATCGATGTGGGAATAA

### Stable cell line generation

To generate stable shRNA knockdown cells, lentiviruses were generated in 293T using the PLKO.1 lentivirus packaging system with gene-specific shRNAs. After 48 hours of lentivirus production, the media was collected and cell debris was removed using a 0.45 um microfiltration membrane. The lentivirus was immediately added to cells in a 6-well dish, along with 2 μg/ml polybrene. The cells were then diluted 1/20 and transferred to a 10 cm^2^ dish 24 hours after transduction and analyzed by RT-qPCR to confirm knockdown.

### Cell proliferation assays

CCK-8 (beyotime, Cell Counting Kit-8) was added to complete culture medium of the same volume, cultured for the same duration as the experimental group, and the absorbance at 450nm was measured together. Cells in logarithmic growth phase with good condition were selected to prepare cell suspension and counted. Approximately 100μl of cell suspension was seeded per well based on appropriate cell seeding density, with 4-6 replicate wells per group. The culture plate was pre-incubated in a cell culture incubator (37°C, 5% CO_2_) for 12-24 hours to allow cells to reach the logarithmic growth phase. 10μl of CCK-8 reagent was added to each well. The culture plate was returned to the cell culture incubator and further incubated for 0.5-4 hours. The absorbance at 450nm wavelength (OD value) was measured using a microplate reader. The experiment was repeated three times, and the average of the experimental results was taken as the final experimental result. Cell viability (%) = [(As-Ab) / (Ac-Ab)] × 100%; As: Absorbance of the experimental group (including cells, culture medium, CCK-8 solution, and drug solution); Ac: Absorbance of the control group (including cells, culture medium, CCK-8 solution, without drug); Ab: Absorbance of the blank group (including culture medium, CCK-8 solution, without cells or drug).

### RNA isolation and Real-time PCR

Total RNA was extracted from the samples using TRIzol (Vazyme, Shanghai), following the instructions provided by the manufacturer. Subsequently, cDNA was synthesized using the Reverse Transcript Kit (Vazyme). Real-time PCR was then carried out in triplicate using the SYBR Green Master Mixture (Vazyme) on the Real-time PCR Detection System (Roche). Quantification was determined based on the cycle threshold (Ct) value and calculated using the 2^-ΔΔCt^ method. The primer sequences are listed below:

MTHFD2-forward: ACTCCCAGAGCACATTGATG

MTHFD2-reverse: CCAGCCACTACCACATTCTT

VEGF-forward: TCAAACCTCACCAAAGCCAG

VEGF-reverse: TCTGAACAAGGCTCACAGTG

P21-forward: ACATCTCAGGGCCGAAAAC

P21-reverse: TGGAGACTGGGAGAGGG

### Preparation of cells for flow cytometry

Cells were seeded according to experimental requirements, and cells were harvested when they reached the desired density. The original culture medium was collected into centrifuge tubes, 1×trypsin digestion was added, and digestion was stopped by adding the original culture medium after the specified time, followed by centrifugation. The supernatant was removed, and the cells were resuspended in 1ml pre-chilled PBS buffer and transferred to 1.5ml Eppendorf tubes, then centrifuged at 4°C, 1000g for 5 minutes. The supernatant was removed, leaving approximately 50μl, gently tapping the bottom of the tube to loosely separate the cells. The dispersed cell suspension was added to 1ml pre-chilled 70% ethanol, gently mixed by pipetting, and fixed at 4°C for at least 4 hours in the refrigerator. The fixed cells were removed, centrifuged at 4°C, 1000g for 5 minutes, the supernatant was removed, 1ml pre-chilled PBS buffer was added to resuspend the cells, followed by centrifugation, removal of the supernatant, leaving 50μl PBS buffer, and gently tapping the tube bottom to separate the cells. Dye preparation: Dyes were prepared according to the instructions based on the number of samples, with the entire process conducted in the dark. 500μl propidium iodide staining solution was added to each sample tube, mixed slowly with a pipette gun, incubated in the dark at 37°C for 30 minutes, after completion, data was saved using a flow cytometer, and subsequent processing was carried out.

### Western blot analysis

We electrophoresed equal amounts of lysates, ranging from 30 to 50 μg, onto polyvinylidene difluoride membranes. Subsequently, the membranes were blocked using PBST with 5% milk and probed with primary antibodies, specifically Actin (1:3000; Proteintech, P62736), and P21 (1:1000, Proteintech, P63000), overnight at 4°C. After washing thrice with PBST, the membranes were incubated for 1 h at room temperature with secondary antibodies, including goat anti-rabbit IgG-HRP (1:4000, Proteintech, SA00001-2) and goat anti-mouse IgG-HRP (1:2000, Proteintech, SA00001-1).

### Flow cytometry

The stained cells were analyzed and sorted based on DNA-A and DNA-W of the Sytox Green fluorescence signal, as well as FSC and SSC light scattering. The analysis was conducted using an LSRII flow cytometer (Becton Dickinson, San Jose, CA, U.S.A.) with an excitation wavelength of 488 nm. The cells were sorted using a FACS Digital Vantage PE flow cytometer (Becton Dickinson) with the same excitation wavelength. The selected channels are as follows: Alexa Fluor™488: Excitation at 488 nm, emission collected in the 530/30 band; PI (Propidium Iodide): Excitation at 561 nm, emission collected in the 610/20 band.

### Animal experimentation

Seven-week-old male wild-type C57BL/6 mice were kept in a controlled environment with a 12-hour light/dark cycle, ensuring a consistent temperature and pathogen-free conditions. They had free access to food and water. The mice were sacrificed either when the tumor size reached 200 mm^2^ or when clear signs of discomfort were observed, in accordance with the guidelines established by the Institutional Animal Care and Use Committee of Tongji University Cancer Center, Shanghai Tenth People's Hospital, School of Medicine, Tongji University (22KN151).

### Statistical analysis

This study independently repeated all experiments three times or more, and all data were analyzed using GraphPad Prism 8. The data are presented as mean ± SEM. Statistical differences were tested using one-way ANOVA, two-way ANOVA. A significance level of *P* < 0.05 indicates statistical significance. In the Figures, *P < 0.05, **P < 0.01, ***P < 0.001, ****P < 0.0001, and "ns" indicates no statistical significance.

## Figures and Tables

**Table 1:** Clinical information of breast cancer patients

Variable	Total	No recurrence	Recurrence	P Value
Age at diagnosis, years				*P* < 0.0001
≤ 60	989	879	110	
> 60	266	209	57	
Histopathological grade				*P* < 0.001
Ⅰ	56	55	1	
Ⅱ	363	307	56	
Ⅲ	176	165	11	
T stage				*P* = 0.42
T1+T2	765	693	72	
T3+T4	55	48	7	
N stage				*P* < 0.0001
N0	517	477	40	
N1+N2+N3	96	57	39	

## Abbreviations

SASP: Senescence-associated secretory phenotype
MTHFD2: Methylenetetrahydrofolate dehydrogenase 2
SCNAs: Somatic copy number alterations
MAF: Mutation Annotation Format
GDC: Genomic Data Commons
TMB: Tumor Mutation Burden
DEGs: Differentially expressed genes
FDR: False discovery rate
GO: Gene Ontology
KEGG: Kyoto Encyclopedia of Genes and Genomes
GSEA: Gene set enrichment analysis
scRNA-seq: Single-cell RNA sequencing
MNN: Mutual nearest neighbor
UMAP: Uniform manifold approximation and projection
CAFs: Cancer-associated fibroblasts
